# Effect of functional genetic variants in chemokine decoy receptors on the recurrence risk of breast cancer

**DOI:** 10.1002/cam4.1823

**Published:** 2018-10-24

**Authors:** Dou‐Dou Li, Chen Yang, Zhi‐Ming Shao, Ke‐Da Yu

**Affiliations:** ^1^ Department of Breast Surgery, Shanghai Cancer Center and Cancer Institute, Shanghai Medical College Fudan University Shanghai China; ^2^ Department of Medical Oncology, Ruijin Hospital Shanghai Jiao Tong University Shanghai China

## Abstract

Duffy antigen receptor for chemokine (DARC) and CCBP2, the two members of chemokine decoy receptor family, restrain cell proliferation and invasion through sequestrating cytotoxic chemokines. Our previous research clarified two functional nonsynonymous single nucleotide polymorphisms (SNPs): rs12075 in DARC and rs2228468 in CCBP2 were significantly correlated with lymph node metastasis. However, the role of their genetic variations on survival of breast cancer remains unclear. In the present study, rs12075 in DARC and rs2228468 in CCBP2 were genotyped in 806 patients with primary breast cancer. The endpoint was recurrence‐free survival (RFS). Cox regression model was used to explore the association between SNPs and patients’ survival. The results revealed that participants with GG genotype in rs12075 appeared a higher recurrence risk compared with AG/AA genotype after adjustment with clinical parameters including lymph node status (AG+AA vs GG: hazard ratio [HR] = 0.54, 95% confidence interval [CI], 0.31‐0.93, *P* = 0.027). Furthermore, subgroup analysis revealed that GG genotype frequency of rs12075 had a positive correlation with RFS compared with AG/AA genotype (AG+AA vs GG: HR = 0.22, 95% CI, 0.05‐0.91, *P* = 0.021) in triple‐negative breast cancer (TNBC) subtype but not in other subtypes. No significant association between the genotypic variants and relapse risk was found in rs2228468 (AC+AA vs CC: HR = 0.80, 95% CI, 0.56‐1.14, *P* = 0.222). There was also no significant difference in survival among rs2228468 polymorphism in any subtypes. Our study suggested that rs12075 could be served as a key predictive factor of recurrence risk in breast cancer, especially for TNBC subtype. Further researches to monitor SNPs will provide further opportunities to determine clinical prognosis.

## 1 INTRODUCTION

Breast cancer, as the most prevalent diagnosed malignancy in female, is a heterogeneous multifactorial disease that attributes to complex interactions between genetic and environmental factors.^1^, ^2^ Although previous studies have identified women who carry a certain genetic variant response for its susceptibility in various populations, there remain a large proportion of treatment failure and mortality subsequently resulting from relapse and metastasis.^3^ Therefore, a better understanding of genetic determinants that predict breast cancer relapse may lead to the development of novel therapeutic strategies to improve patients’ outcomes.

Chemokine and chemokine receptors are believed to promote tumor progression, stimulate angiogenesis, and induce epithelial‐mesenchymal transition.^4^, ^5^, ^6^, ^7^ Emerging evidence indicates that breast cancer progression and metastasis is attributable to chemokine and chemokine receptors.^8^, ^9^ Chemokine decoy receptors (CDR), as a new subgroup of chemokine receptors , cast as scavengers by efficiently internalizing their cognate chemokine ligands.^10^ Recent studies have suggested that Duffy antigen receptor for chemokine (DARC) and CCBP2, the two representative members of CDR family, act as physical barrier to the sequestration of cytotoxic chemokines to restrain cancer cell proliferation and invasion in breast cancer.^11^, ^12^ Furthermore, DARC and CCBP2 have been found abundantly expressed on lymphatic and hematogenous cells which play an essential role in inhibiting metastasis.^13^, ^14^, ^15^ Gene polymorphisms in promoter regions of chemokines provide valuable linkage for the susceptibility to malignant diseases. In humans, low expression of DARC was associated with increased lymph node and distant metastasis and outcomes in breast cancer. CCBP2 was served as a checkpoint for neutrophil release and antimetastatic activity. Previous research in our laboratory has clarified the two nonsynonymous SNPs: rs12075 (G42D) and rs2228468 (S373Y). rs12075 in DARC and rs2228468 in CCBP2 were significantly correlated with lymph node metastasis in a dominant model, but not in a recessive model,^16^ which manifested that genetic polymorphisms in the genes encoding CDRs could mediate metastatic risk. However, the role of CDR in genetic variations on survival prognosis of breast cancer remains unclear.

In view of the broad distribution of the two potentially functional nonsynonymous single nucleotide polymorphisms (SNPs) as well as the capability of decreasing the possibility of lymph node metastasis, we hypothesized that carrying different levels of CDR genetic variants and genotyping might affect the long‐term survival of breast cancer. In this study, we investigated the survival effects of genetic variations of rs12075 and rs2228468 in a cohort of patients with primary breast cancer. Besides, we first illuminated the correlation of recurrence‐free survival (RFS) with different molecular subtypes in the participants with a long follow‐up. Our research attempted to seek a promising recurrence predictor which could optimize more appropriate therapeutic measures.

## 2 METHODS

### 2.1 Ethics statement

This study was approved by the Research Ethics Committee of Shanghai Cancer Center of Fudan University. Written informed consents were obtained from all the participants.

### 2.2 Study population

From 2006 to 2008, a total of 833 female patients with pathologically confirmed operable primary invasive breast cancer from Shanghai Cancer Center were enrolled in the present study. Subjects were identified as genetically unrelated Han descent. Participants who selected for the analysis should meet the following inclusion criteria: (a) underwent mastectomy or lumpectomy plus level I/II axillary lymph node dissection or sentinel node biopsy; (b) pathologically and histologically confirmed invasive ductal breast cancer at department of pathology of Fudan University Shanghai Cancer Center; ductal carcinoma in situ (with or without microinvasion) was excluded; (c) no receipt of neoadjuvant therapy (including chemotherapy, radiotherapy or hormone therapy); (d) unilateral breast cancer; (e) no any history of other cancers; and (f) at least 2 months of follow‐up data. Among them, 27 cases were excluded because of genotyping failure. Therefore, 806 patients were included in the final analysis.

Pathologic examination of the lymph nodes was identified through hematoxylin and eosin (H&E) staining. The estrogen receptor (ER), progesterone receptor (PR), and human epidermal growth factor receptor 2 (HER2) statuses were confirmed by immunohistochemical staining. Positive ER or PR required equal or more than 10% of tumor cell immune responses. Patients with equal HER2 protein expression (immunohistochemistry 2+) were selected to have a fluorescent in situ hybridization (FISH) test for HER2 gene amplification. This is carried out in accordance with standard procedures. Because ki67 data were partly missing, we modified the molecular subtypes of breast cancer as follows: luminal A = ER+ or PR+, and HER2−; luminal B = ER+ or PR+, and HER2+; HER2‐enriched (HER2+) = ER−, PR−, and HER2+; and TNBC = ER−, PR−, and HER2−. Clinicopathological characteristics were extracted from the patients’ medical documents.

### 2.3 Single nucleotide polymorphisms selection and genotyping

Selection of genetic variants was described in detail in our previous study.^16^ We identified rs12075 through screening polymorphisms across the DARC and CCBP2 genetic region and its flanking sequences by directly sequencing the PCR products of genomic DNAs from the blood samples of 24 patients with sporadic breast cancer. The two SNPs were further genotyping, which were carried out by the Chinese National Human Genome Center (Shanghai) as well as using the 12‐plex SNP stream system. The sequences of the primers for each SNP are listed in Table S1. To confirm the genotyping results, 10% of the DNA samples were randomly selected for direct sequencing, and the results were 100% concordant.

### 2.4 Statistical analysis

Statistical analyses were performed using SPSS 22.0 for Windows (IBM, Armonk, NY, USA). The means and standard deviations (SDs) were calculated for age variable, and percentages were calculated for clinicopathological variables. RFS was measured from the date of surgery to the date of first local/regional recurrence or distant metastasis. Survival curves were constructed by the Kaplan‐Meier method, and the difference was detected by log‐rank test. Because the AA genotype presented in four cases, for minimizing the error, we combined AA and AG for further analysis. The effects of each clinicopathological data and SNP genotypes on RFS were used by the univariate and multivariate Cox regression, estimating hazard ratio (HR), and 95% confident interval (CI). Clinicopathological factors with *P*‐values of 0.10 in univariate Cox analysis were enrolled in the multivariate Cox model. All tests performed were two‐sided. Differences were considered statistically significant if *P* < 0.05.

## 3 RESULTS

### 3.1 Clinicopathological characteristics of study population

A total of 806 patients enrolled in this study. The median follow‐up time was 48 months. Demographic distributions and clinicopathologic characteristics of breast cancer patients are summarized in Table 1. The mean age of enroll patients at the time of diagnosis was 49.0 ± 12.0 years. All the patients were diagnosed invasive ductal carcinoma with 53.6% had early‐stage tumor (T1). Additionally, 42.8% exhibited lymph node involvement. Most of the cases harbored luminal A (58.3%) subtype, and luminal B and HER2‐positive account for 11.8% and 10.8, respectively. During the follow‐up period, 130 patients developed recurrence. Similar to the data from HapMap database for the Han Chinese population, the genotype frequencies of rs12075 showed GG 85.7%, AG 13.8%, and AA 0.5%, and the frequencies of rs2228468 were CC 43.8%, AC 45.9%, and AA 9.3%, respectively. No significant difference in genotype frequencies from the Hardy‐Weinberg equilibrium test was observed for the two SNPs (both *P* values were >0.05).

**Table 1:** Clinicopathological characteristics of the breast cancer patients

Characteristics	Patients n (%)
Mean age (±SD)	
49.0 ± 12.0	
Age (y)	
<50	398 (49.4)
≥50	408 (50.6)
Menopausal status	
Premenopausal	456 (56.6)
Postmenopausal	350 (43.4)
Tumor size (cm)	
≤2	432 (53.6)
>2	352 (43.7)
Lymph node status	
Positive	345 (42.8)
Negative	455 (56.5)
ER status	
Positive	517 (64.1)
Negative	289 (35.9)
PR status	
Positive	458 (56.8)
Negative	348 (43.2)
HER2 status	
Positive	182 (22.6)
Negative	624 (77.4)
Molecular subtype	
Luminal A	470 (58.3)
Luminal B	95 (11.8)
HER2+	87 (10.8)
Triple negative	154(19.1)
Adjuvant chemotherapy	
Yes	580 (72.0)
No	226 (28.0)
Endocrine therapy	
Yes	544 (67.5)
No	248 (30.8)
SNP rs12075	
GG	691 (85.7)
AG	111 (13.8)
AA	4 (0.5)
SNP rs2228468	
CC	353 (43.8)
AC	370 (45.9)
AA	75 (9.3)

ER, estrogen receptor; HER2, human epidermal growth factor receptor 2; PR, progesterone receptor.

### 3.2 Association analysis of SNPs with RFS in breast cancer

**Figure 1:**
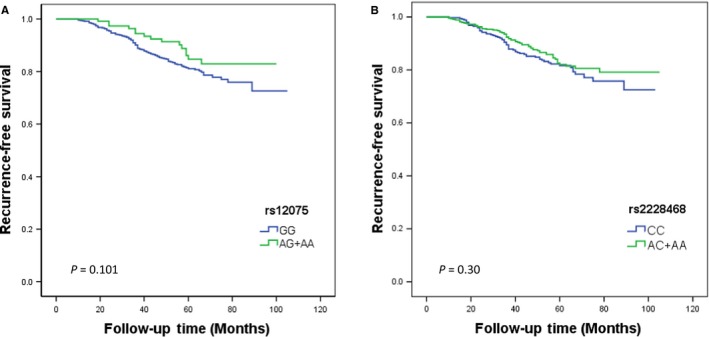
Effects of rs12075 and rs2228468 genotypes on RFS. Kaplan‐Meier estimates of RFS in 806 breast cancer patients according to the (A) rs12075 and (B) rs2228468, and the survival differences between groups were compared by log‐rank test

We conducted univariate analysis to evaluate the prognostic effects of all selected characteristics on RFS by Cox regression model. As shown in Table 2, we investigated the genotypic association between the two SNPs and breast cancer risk in a dominant model, and the results revealed that GG genotype frequency of rs12075 polymorphism revealed an association with high risk of breast cancer (dominant model: AG+AA vs GG: HR = 0.64, 95% CI: 0.37‐1.10, *P* = 0.10). For rs2228468, no significant association was found between the genotypic variants and breast cancer in dominant model (dominant model: AC+AA vs CC: HR = 0.83, 95% CI: 0.59‐1.18, *P* = 0.30). After adjustment with tumor size, lymph node status, ER, PR, HER2, and endocrine therapy, we founded that participants with GG genotype appeared a higher recurrence risk compared with patients with AG or AA genotype, which indicated that rs12075 was a significant prognostic marker under dominant models (AG+AA vs GG: HR = 0.54, 95% CI: 0.31‐0.93, *P* = 0.027). However, for rs2228468, multivariate analysis indicates that the RFS rate for the CC genotype was similar to the AG or AA genotype (AC+AA vs CC: HR = 0.80, 95% CI: 0.56‐1.14, *P* = 0.222). In conclusion, it suggests that the breast cancer patients with GG genotypes of rs12075 exhibit for a worse RFS. As shown in Figure 1, Kaplan‐Meier curves indicated a tendency toward detrimental to survival in the patients with the GG genotype of rs12075 compared to AG or AA genotype (*P* = 0.10). For rs2228468, the patients who carried the CC genotype had statistically insignificant poorer prognosis than those with AC or AA genotype (*P* = 0.30).

**Table 2:** Univariate and multivariate Cox regression analysis of RFS for clinical risk factors and SNP rs12075 and rs2228468 in breast cancer patients

Parameters	Univariate Cox regression analysis		Multivariate Cox regression analysis	
	HR (95% CI)	*P*	HR (95% CI)	*P*
Age (y) (<50 vs ≥50)	1.24 (0.83‐1.86)	0.55	—	—
Menopausal status (Pre. vs Post.)	1.01 (0.72‐1.44)	0.94	—	—
Tumor size (cm) (<5 vs ≥5)	1.85 (1.30‐2.63)	<0.001	1.51 (1.05‐2.17)	0.026
Lymph node status (Neg. vs Pos.)	2.21 (1.56‐3.14)	<0.001	1.99 (1.38‐2.86)	0.001
ER status (Neg. vs Pos.)	0.41 (0.29‐0.59)	<0.001	0.40 (0.22‐0.75)	0.004
PR status (Neg. vs Pos.)	0.43 (0.30‐0.62)	<0.001	0.59 (0.34‐1.02)	0.057
HER2 status (Neg. vs Pos.)	1.95 (1.37‐2.78)	<0.001	1.59 (1.08‐2.33)	0.018
Adjuvant chemotherapy (No vs Yes)	1.24 (0.83‐1.89)	0.29	—	—
Endocrine therapy (No vs Yes)	0.45 (0.32‐0.63)	<0.001	1.77 (0.82‐3.82)	0.147
rs12075 (GG vs AG+AA)	0.64 (0.37‐1.10)	0.10	0.54 (0.31‐0.93)	0.027
rs2228468 (CC vs AC+AA)	0.83 (0.59‐1.18)	0.30	0.80 (0.56‐1.14)	0.222

CI, confidence interval; ER, estrogen receptor; HER2, human epidermal growth factor receptor 2; HR, hazard ratio; PR, progesterone receptor.

### 3.3 Stratification analysis of breast cancer according to different molecular subtypes

**Figure 2:**
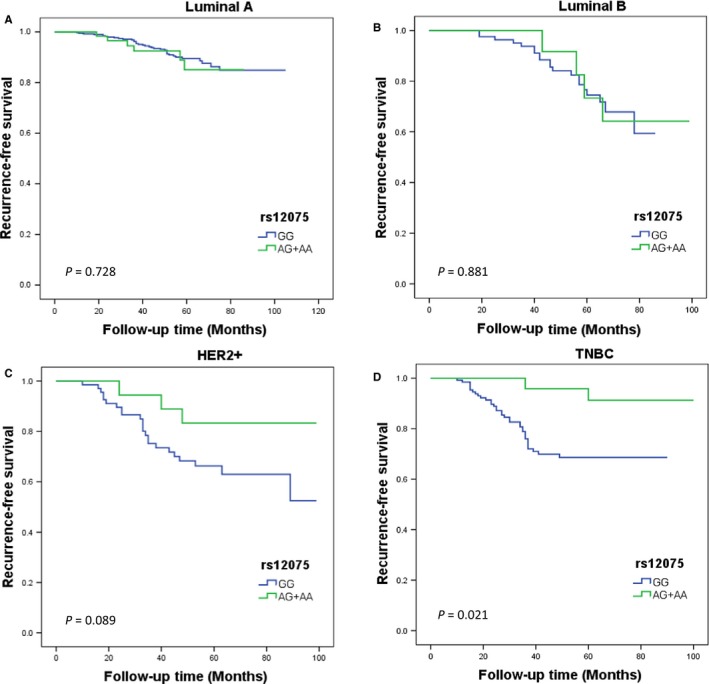
Kaplan‐Meier estimates of rs12075 genotypes on RFS stratified by different molecular subtypes: (A) luminal A, (B) luminal B, (C) HER2+, and (D) TNBC. The survival differences between groups were compared by log‐rank test

**Figure 3:**
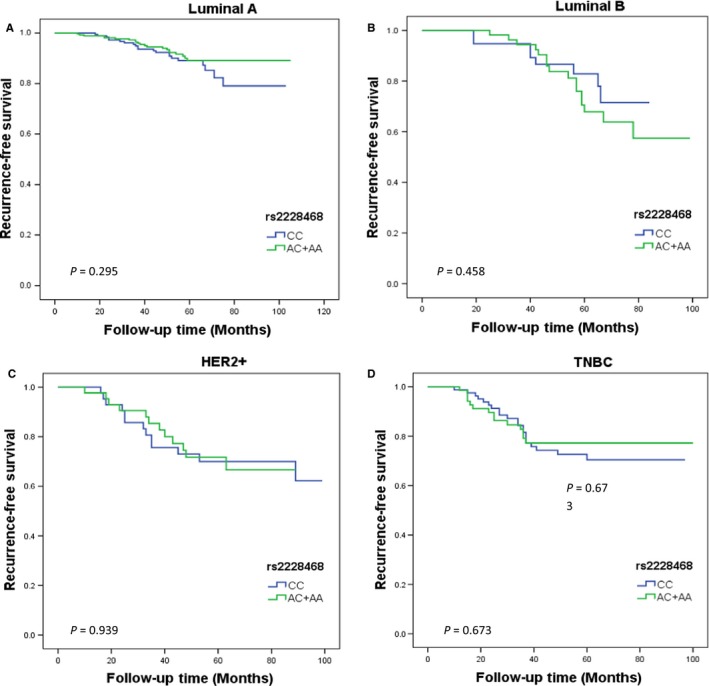
Kaplan‐Meier estimates of rs2228468 genotypes on RFS stratified by different molecular subtypes: (A) luminal A, (B) luminal B, (C) HER2+, and (D) TNBC. The survival differences between groups were compared by log‐rank test

Our data showed that the RFS time of participants carrying GG genotype of rs12075 was apparently lower than that of patients carrying AG or AA genotype in TNBC subtype (*P* = 0.021), but not for other three subtypes (*P* = 0.728 for luminal A, *P* = 0.881 for luminal B, and *P* = 0.089 for HER2+; Figure 2). After adjusting for lymph node status and tumor size, the result remained borderline statistically significant association of RFS with this SNP for the TNBC subtype (AG+AA vs GG: HR = 0.28, 95% CI: 0.07‐1.17, *P* = 0.080). In contrast, for the rs2228468, there was also no evidence of significant difference in survival rate in any subtypes (Figure 3). Similarly, further multivariate analysis remained no significant association between the different genotypes of rs2228468 polymorphisms and RFS in any molecular subtypes (Table 3).

**Table 3:** Univariate and multivariate Cox regression analysis of rs12075 and rs2228468 in different molecular subtypes after adjusting for lymph node status and tumor size

	Univariate Cox regression analysis		Multivariate Cox regression analysis	
	HR (95% CI)	*P*	HR (95% CI)	*P*
Luminal A				
rs12075 (GG vs AG+AA)	1.17 (0.49‐2.77)	0.728	0.96 (0.40‐2.31)	0.932
rs2228468 (CC vs AC+AA)	0.73 (0.40‐1.33)	0.295	0.73 (0.40‐1.34)	0.316
Luminal B				
rs12075 (GG vs AG+AA)	0.92 (0.31‐2.73)	0.881	0.74 (0.24‐2.24)	0.602
rs2228468 (CC vs AC+AA)	1.38 (0.59‐3.23)	0.458	1.28 (0.54‐3.00)	0.575
HER2+				
rs12075 (GG vs AG+AA)	0.37 (0.11‐1.23)	0.089	0.35 (0.10‐1.20)	0.096
rs2228468 (CC vs AC+AA)	0.97 (0.44‐2.14)	0.939	0.89 (0.40‐1.97)	0.772
TNBC				
rs12075 (GG vs AG+AA)	0.22 (0.05‐0.91)	0.021	0.28 (0.07‐1.17)	0.080
rs2228468 (CC vs AC+AA)	0.87 (0.44‐1.70)	0.673	0.61 (0.29‐1.25)	0.173

CI, confidence interval; ER, estrogen receptor; HER2, human epidermal growth factor receptor 2; HR, hazard ratio; PR, progesterone receptor; TNBC, triple‐negative breast cancer.

Multivariate Cox regression analysis Adjusted for lymph node status, tumor size.

## 4 DISCUSSION

In this study, we recruited patients with breast cancer and uncovered the predictive value of the two SNP genotypes incorporated with clinicopathologic factors. In line with our expectations, we demonstrated that GG genotype frequency of rs12075 polymorphism revealed an association with high risk of breast cancer along with a tendency toward poor survival after including adjustment with clinicopathologic elements. We also found rs12075 carrying GG genotype developed vulnerability to relapse in TNBC subtype compared to other three molecular subtypes. Nevertheless, no significant prediction effect of rs2228468 with different genotypes was detected even if some types prone to develop lymph node metastasis. Similarly, there was no obvious difference between SNP genotypes and the four common molecular subtypes in rs2228468. Collectively, our findings revealed that rs12075 polymorphism represented an attractive means by which to enhance the effectiveness of radiotherapy through predicting recurrence in breast cancer.

Although improvements come forth in constantly update and optimization of therapies, their benefits for breast cancer remain limited on account of recurrence and metastasis. The identification of genetic polymorphisms including SNPs may facilitate the development of identifying individuals at high risk of breast cancer and can be leveraged to explore new therapeutic strategies. Recent studies have highlighted chemokines and their receptors as more notable role in tumor environment stabilization as well as recurrence and metastasis.^17^, ^18^ Thus, pro‐malignant chemokine concentrations regulated by SNPs in the genes encoding CDRs may closely relate to breast cancer metastasis.

DARC as a silent chemokine receptor, along with CCBP2 and the CCXCKR, comprises CDR family. Recently, studies highlight that DARC plays a potential role in malignancy, the most essential of which is served as a inhibit barrier of metastases.^19^, ^20^ Studies increasingly discovered the association between clinical outcomes and DARC: Necrosis and decreased metastases were induced by DARC in lung cancer; the absence of DARC expression in prostate cancer tended to poor survival; and the expression of DARC by epithelial ovarian cancer decreases growth potential.^11^, ^21^, ^22^, ^23^ Multiple reports showed that downregulation of CCBP2 in transformed cells was consistent with tumor progression and oncogene activation in Kaposi sarcoma.^24^ Accordingly, genetic inactivation of CCBP2 unleashes metastatic potential.^25^ In view of the influence on LNM mediated by the two SNPs in our early report, which only probed into the relationship between LNM and the two SNPs owed to follow‐up time limitation. In the present study, we thoroughly investigated the prognostic value of rs12075 and rs2228468 in breast cancer.

In agreement with the above data, several limitations must be taken into account. The main limitation is considered to be the insufficient follow‐up time. On the basis of the Kaplan‐Meier curves, there remains more than 50% of the patients survived at the end of the follow‐up; thus, the outcomes seem less rigorous. Moreover, uneven distribution of genotypes in patients comes up with a relative basis outcome. As far as the genotypes in rs12075, AA genotype only presents in four cases. All of which may not be sufficient to explain a difference in outcomes. Furthermore, in an effort to strengthen and extend these findings, the need for more eligible patients and further research with more survival prognosis outcomes should be launched to confirm prognosis effectiveness of the two SNPs.

In conclusion, our study has elucidated that SNP rs12075 serves as a key predictive factor of recurrence risk in postoperate breast cancer patients, especially for TNBC subtype. Further researches to monitor SNPs in large sample sets in combination with comprehensive clinicopathologic databases will provide further opportunities to determine clinical prognosis. Corresponding measures may pave the way for the innovative therapeutic strategies for reducing recurrence rate.

## ETHICAL APPROVAL

All procedures performed in studies involving human participants were in accordance with the ethical standards of the institutional and/or national research committee and with the 1964 Helsinki Declaration and its later amendments or comparable ethical standards.

## CONFLICT OF INTEREST

All the authors declare no conflict of interest.

## INFORMED CONSENT

Informed consent was obtained from all individual participants included in the study.
