# Supplementary material for: Effect of functional genetic variants in chemokine decoy receptors on the recurrence risk of breast cancer
Source: Cancer Med. 2018 Oct 24;7(11):5497–504. doi: 10.1002/cam4.1823 (PMC6247033; doi:10.1002/cam4.1823)
Supplement: Supplementary file 1 [file CAM4-7-5497-s001.docx]

**Table S1 Primers and probes for rs12075 and rs2228468**

| SNP | Primers and Probes | |
| --- | --- | --- |
| rs12075 | Up | TGGACTTCGAAGATGTATGGA |
|  | Down | AGAGTCATCCAGCAGGTTACAG |
|  | Probe | CGACTGTAGGTGCGTAACTCGATTCCTTCCCAGATGGAGACTATG |
| rs2228468 | Up | TTCCCACCAGACCAAAATT |
|  | Down | ATTATCCAGCTGTTCTGAGAGC |
|  | Probe | GGCTATGATTCGCAATGCTTATTCCCCACATCCTCCTTGTTAGGG |
